# Expression Profiling in Ovarian Cancer Reveals Coordinated Regulation of *BRCA1/2* and Homologous Recombination Genes

**DOI:** 10.3390/biomedicines10020199

**Published:** 2022-01-18

**Authors:** Noélia Custódio, Rosina Savisaar, Célia Carvalho, Pedro Bak-Gordon, Maria I. Ribeiro, Joana Tavares, Paula B. Nunes, Ana Peixoto, Carla Pinto, Carla Escudeiro, Manuel R. Teixeira, Maria Carmo-Fonseca

**Affiliations:** 1 Instituto de Medicina Molecular João Lobo Antunes, Faculdade de Medicina, Universidade de Lisboa, 1649-028 Lisboa, Portugal; rsavisaar@medicina.ulisboa.pt (R.S.); hcelia@medicina.ulisboa.pt (C.C.); gordonpedro@campus.ul.pt (P.B.-G.); mifr@campus.ul.pt (M.I.R.); carmo.fonseca@medicina.ulisboa.pt (M.C.-F.); 2 Serviço de Anatomia Patológica, Hospital de Santa Maria, Centro Hospitalar Universitário Lisboa Norte, 1649-028 Lisboa, Portugal; joana.almeida@chln.min-saude.pt; 3 Hospital CUF Descobertas, 1998-018 Lisboa, Portugal; paula.b.nunes@cuf.pt; 4 Faculdade de Medicina, Universidade de Lisboa, 1649-028 Lisboa, Portugal; 5 Serviço de Genética, Instituto Português de Oncologia do Porto Francisco Gentil, 4200-072 Porto, Portugal; analuisamoura@ipoporto.min-saude.pt (A.P.); carla.a.pinto@ipoporto.min-saude.pt (C.P.); carla.escudeiro@ipoporto.min-saude.pt (C.E.); manuelteixeira@ipoporto.min-saude.pt (M.R.T.)

**Keywords:** ovarian cancer, *BRCA1/2* mRNA, homologous recombination, nCounter assay, ddPCR

## Abstract

Predictive biomarkers are crucial in clarifying the best strategy to use poly(ADP-ribose) polymerase inhibitors (PARPi) for the greatest benefit to ovarian cancer patients. PARPi are specifically lethal to cancer cells that cannot repair DNA damage by homologous recombination (HR), and HR deficiency is frequently associated with *BRCA1/2* mutations. Genetic tests for *BRCA1/2* mutations are currently used in the clinic, but results can be inconclusive due to the high prevalence of rare DNA sequence variants of unknown significance. Most tests also fail to detect epigenetic modifications and mutations located deep within introns that may alter the mRNA. The aim of this study was to investigate whether quantitation of *BRCA1/2* mRNAs in ovarian cancer can provide information beyond the DNA tests. Using the nCounter assay from NanoString Technologies, we analyzed RNA isolated from 38 ovarian cancer specimens and 11 normal fallopian tube samples. We found that *BRCA1/2* expression was highly variable among tumors. We further observed that tumors with lower levels of *BRCA1/2* mRNA showed downregulated expression of 12 additional HR genes. Analysis of 299 ovarian cancer samples from The Cancer Genome Atlas (TCGA) confirmed the coordinated expression of *BRCA1/2* and HR genes. To facilitate the routine analysis of *BRCA1/2* mRNA in the clinical setting, we developed a targeted droplet digital PCR approach that can be used with FFPE samples. In conclusion, this study underscores the potential clinical benefit of measuring mRNA levels in tumors when *BRCA1/2* DNA tests are negative or inconclusive.

## 1. Introduction

Ovarian cancer is the deadliest form of gynecological cancer [1,2]. The average lifetime risk of developing ovarian cancer is 1.3%, the equivalent of 1 in 78 women [1]. Although ovarian cancer accounts for 2.5% of all malignancies in women, it is responsible for 5% of female cancer deaths [2]. This high fatality rate is usually attributed to frequent late diagnosis, limited therapeutic options, and a lack of effective biomarkers for predicting treatment response [3]. 

The standard treatment for newly diagnosed ovarian cancer is cytoreductive surgery followed by platinum-based chemotherapy [4]. However, approximately 70% of patients have a relapse within the subsequent 3 years [3]. Recurrent ovarian cancer is typically incurable, with most patients receiving multiple additional lines of treatment. 

Over the past 10 years, a new class of small molecule drugs that inhibit poly(adenosine diphosphate-ribose) polymerases (PARPs) has attracted much attention for the treatment of ovarian cancer [5]. The PARP family comprises several proteins involved in a variety of cellular processes, including the stress response, chromatin remodeling, DNA repair, and apoptosis [6]. One of the best-characterized targets of PARP inhibitors (PARPi) is PARP1, a protein that plays a major role in the detection and repair of single-strand DNA breaks [6,7]. PARP1 is also likely involved in other DNA repair pathways, such as nucleotide excision repair, non-homologous end joining, homologous recombination, and DNA mismatch repair [6,7]. Mechanistically, PARP1 is a DNA damage sensor and a signal transducer that binds to DNA breaks and then synthesizes poly(ADP-ribose) chains on target proteins (PARylation), including PARP1 itself (autoPARylation). Auto-PARylation further activates PARP1 and enables the PARylation of histones and other chromatin-associated proteins, which leads to the recruitment of additional DNA repair molecules to the site of damage [7].

In 2005, two studies revealed that tumor cells lacking *BRCA1* or *BRCA2* proteins are selectively vulnerable to PARPi [8,9]. Inhibition of PARP1 alone is not lethal for normal cells, but in the absence of *BRCA1/2*, it becomes cytotoxic for tumor cells [8,9]. The proposed mechanism of action is that PARP1 inhibition prevents PARylation and locks PARP1 on damaged DNA, stalling the progress of replication forks. In dividing cells treated with PARPi, stalled replication forks can be rescued by homologous recombination (HR), a process that enables high-fidelity, template-dependent repair of DNA damage [10]. Tumor cells that are defective in HR due to loss of *BRCA1* or *BRCA2* use alternative error-prone pathways that lead to fragmentation of the genome and ultimately result in cell death [5]. In addition to *BRCA1* and *BRCA2*, several other proteins are involved in the HR process [11], and tumors with BRCA-independent HR deficiency are also sensitive to PARPi [6]. Although the first approved PARPi (olaparib) initially targeted patients with ovarian cancer that carried germline mutations in the *BRCA1* or *BRCA2* genes, a later study demonstrated responses with olaparib in 41% and 24% of germline BRCA mutation carriers and non-carriers, respectively [12]. Among the non-carriers, a sub-set had somatic BRCA mutations and a few others had mutations in other HR genes, such as *RAD50, RAD51C/D, CDK12,* or *PALB2* [13]. These clinical studies were conducted in patients previously treated with platinum salts, which are DNA damaging agents that cause DNA crosslink repair in part by HR, and revealed a clear association between platinum sensitivity and response to PARPi [14]. Thus, further trials shifted towards using PARPi in patients who responded to platinum-based chemotherapy. Currently, PARPi are approved for the treatment of all platinum-sensitive patients, regardless of *BRCA1/2* status. However, more benefit is seen in patients with either germline or somatic *BRCA1/2* mutations [5].

Inherited susceptibility to ovarian cancer is found in approximately 15% of patients, the vast majority of which have germline heterozygous mutations in the *BRCA1* or *BRCA2* genes [15]. Loss of the wild-type BRCA allele can be detected in cancers that develop in heterozygous carriers, but whether this second hit is required for oncogenesis is unclear [16]. Somatic mutations in *BRCA1* or *BRCA2* genes are also occasionally present in sporadic (non-hereditary) ovarian cancers [15].

Developing predictive biomarkers for PARPi is of critical importance to delivering precision treatment to patients. Currently, DNA tests to detect both germline and somatic mutations in *BRCA1* and *BRCA2* genes are often used in the clinic. However, the utility of such assays is limited by the high prevalence of rare DNA sequence variants of unknown significance (VUS). Indeed, roughly equal numbers of VUS and pathogenic/likely pathogenic variants in *BRCA1/2* are listed in the National Institutes of Health (NIH) repository ClinVar (https://www.ncbi.nlm.nih.gov/clinvar, last accessed on 25 May 2020). Moreover, most currently used DNA assays fail to detect epigenetic modifications (such as promoter hypermethylation), as well as mutations located deep within introns that may alter the mRNA [17].Here, we hypothesized that measuring the level of *BRCA1* and *BRCA2* mRNA in ovarian cancer tissue may provide additional information when DNA tests are negative or inconclusive. Downregulation of *BRCA* mRNA was previously shown to cause dysfunctional expression of BRCA proteins, resulting in HR deficiency and sensitivity to PARPi [18]. Mechanisms leading to reduced mRNA levels include epigenetic silencing and frameshift mutations. In addition to classical frameshift mutations (i.e., insertions, deletions, or duplications of exonic nucleotides that change the reading frame of the mRNA), splicing mutations often induce a frameshift as a consequence of exon skipping, use of unnatural (cryptic) splice sites, or intron retention [17]. Changing the reading frame for translation often introduces a premature termination codon (PTC) into the mRNA, and a cellular surveillance system termed nonsense-mediated mRNA decay (NMD) degrades the vast majority of mRNAs with PTCs [19]. Because NMD leads to the downregulation of expression and loss of function of the affected genes, it contributes to many genetic diseases including cancer. An example of this is the inactivation of tumor suppressor genes harboring PTCs through NMD [20].

The extent to which *BRCA1* and *BRCA2* mRNA levels are regulated in ovarian cancer has remained poorly understood. In this study, we identified a sub-set of tumors lacking any detected *BRCA1/2* mutation that express low levels of *BRCA1/2* mRNA similar to tumors with known NMD-eliciting PTCs. Notably, we found that the expression of 12 other HR genes is also downregulated in tumors with low levels of *BRCA1/2* mRNA. These results underscore the potential clinical benefit of measuring mRNA levels in tumors when *BRCA1*/2 DNA tests are negative or inconclusive. 

## 2. Materials and Methods

### 2.1. Biological Samples

We analyzed archived formalin-fixed and paraffin-embedded (FFPE) tissue biopsies, including ovarian cancer and normal fallopian tube samples. Samples were collected from the archives of the Hospital Santa Maria/Centro Hospitalar Universitário Lisboa Norte, E.P.E., Lisbon, Portugal, and Instituto Português de Oncologia, Porto, Portugal. An experienced pathologist assessed the quality of each sample. In the case of tumor samples, the diagnosis was confirmed, and the tissue was macro dissected to ensure that only tumor regions were further analyzed. The study was approved by the ethical committee of the Lisbon Academic Medical Center, Lisbon, Portugal (approval number: 170/18).

### 2.2. RNA isolation and Quantification

RNA was extracted from 5–8 curls of tissue (10 μm thick) sectioned from each sample. RNA was purified using the PureLink^TM^ FFPE RNA Isolation Kit (, Invitrogen, Carlsbad, CA, USA), as described by the manufacturer. The amount and purity of total RNA recovered were determined with the Nanodrop^TM^ 2000 spectrophotometer (Thermo Scientific, Waltham, MA, USA). Traces of contaminant DNA were removed by DNase I digestion. Finally, RNA concentration and size were analyzed in the Agilent 4200 TapeStation system using an Agilent RNA ScreenTape assay (Agilent, Santa Clara, CA, USA). 

### 2.3. Gene Expression Profiling with the nCounter System

We used the nCounter^®^ FLEX Analysis System and the Vantage 3D™ RNA Panel for DNA Damage and Repair (NanoString Technologies, Seattle, WA, USA). The mRNA hybridization, detection, and scanning were performed at the NanoString nCounter Core Facility at the Serviço de Genética—Instituto Português de Oncologia do Porto Francisco Gentil, E.P.E., Porto, Portugal. Briefly, purified RNA (200 ng) was hybridized overnight at 67 °C. The full volume of the hybridization reactions was immediately introduced in the Prep Station, which is a liquid handling robot that performs the purification of the hybridized complexes and their immobilization onto the surface of a cartridge. The barcodes captured for each sample were then identified and counted by the Digital Analyzer. The resulting data were imported into and analyzed by the nSolver 4.0 Analysis Software System. Quality control parameters recommended by NanoString were applied using the nSolver default settings. Samples that failed the quality control metrics were excluded from further analysis. For data normalization, raw counts were the first background adjusted using the internal negative controls followed by a within-sample normalization using the internal positive controls. Finally, data were normalized across samples (i.e., corrected for input) using the mean RNA counts from reference (housekeeping) genes. This resulted in a total of 49 samples, 11 from normal fallopian tubes and 38 from ovarian cancer tissue. 

### 2.4. BRCA1 and BRCA2 mRNA Analysis by Droplet Digital PCR

The level of *BRCA1* and *BRCA2* mRNA in ovarian cancer samples previously analyzed with the nCounter assay was quantified by droplet digital PCR (ddPCR) using the Bio-Rad QX200 system (Bio-Rad Laboratories, Hercules, CA, USA), according to the manufacturer’s instructions and the digital MIQE guidelines [21,22]. First, purified RNA samples were treated with DNAse I (Roche, Mannheim, Germany) 0.5 U/µL, at 37 °C for 30 min, in the presence of RiboSafe (Bioline, London, UK) 1 U/µL. After purification with an equal volume of phenol:chloroform:isoamyl alcohol (25:24:1) in the presence of 0.3M Sodium acetate pH 5.2 and 50 μg/mL GlycoBlue™ coprecipitant (ThermoFisher Scientific, Waltham, MA, USA), RNA was precipitated with 2 volumes 100% ethanol, dissolved in 0.1 mL of RNAse free water and quantified in NanoDrop 2000 spectrophotometer. Then, RNA was retrotranscribed and amplified using the One-Step RT-ddPCR Advanced Kit for Probes (Bio-Rad, Hercules, CA, USA,) and PrimeTime Std^®^ qPCR Probe Assays from IDT (Integrated DNA Technologies, Coralville, IA, USA). Primers and Taqman fluorescent probes were designed with IDT software tools available online and are described in Supplementary Table S1. All genes were analyzed with 6-FAM/ZEN/IBFQ labeled probes, except reference gene *GUSB* for which we used a HEX/ZEN/IBFQ labeled probe. Duplex experiments were performed targeting either *BRCA1, BRCA2, POLR1E* or *NUBP1* (FAM) and *GUSB* (HEX) simultaneously.

The ddPCR workflow started by partitioning the RT-PCR reaction mix (total volume 20 µL) containing forward and reverse primers (900 nM final concentration), probes (250 nM final concentration each), and sample RNA into aqueous droplets in oil. Negative controls included samples with no reverse transcriptase in the RT-PCR master mix. The droplets were then subjected to a thermocycling protocol for retro transcription and amplification (50 °C for 1 h; 95 °C for 10 min; 46 cycles of 95 °C for 35 s and 60 °C for 1 min; and 98 °C for 10 min). The PCR products were kept at 4 °C for up to 1 day before reading the fluorescent content of the droplets in the QX200 Droplet Reader (Bio-Rad, Hercules, CA, USA). Data were analyzed with the QuantaSoft software (Version 1.7.4.0917, Bio-Rad, Hercules, CA, USA). The fraction of positive and negative droplets was determined based on the fluorescent signal, and the results were fitted to a Poisson distribution to estimate absolute concentrations (copies/µL) of each mRNA. 

To ensure that similar amounts of reference mRNA were loaded per sample, two assays were sequentially performed for each sample. In the first assay, 30 ng of total RNA was loaded and the number of copies per droplet (CPD) of *GUSB* mRNA was quantified. The amount of total RNA loaded in the second assay contained *GUSB* mRNA at a concentration of 0.05 CPD (50 copies/µL).

For comparison, we used the same ddPCR assays to test RNA extracted from non-fixed, freshly isolated peripheral blood mononuclear cells (PBMCs).

### 2.5. Statistical Analysis

#### 2.5.1. General

Analysis and plotting were performed with R Core Team 2019 (version 3.6.1, R Foundation for Statistical Computing, Vienna, Austria). Empirical *p*-values were calculated as $P=\frac{n+1}{m+1}$, where *n* is the number of stimulants that show a value as high as or higher than the true value, and m is the total number of simulants [23], (Spearman’s correlation coefficients were converted to z’-values and back using the functions *FisherZ()* and *FisherZInv()* in the DescTools package v. 0.99.36.

#### 2.5.2. Analysis of NanoString Data

Genes were classified into pathways based on the labels supplied with the NanoString panel. The Homologous Recombination and Fanconi Anemia pathways were grouped, and the relevant genes are referred to as Homologous Recombination (HR) genes throughout the text. Twelve of the genes in the panel (*ERCC1, ERCC2, ERCC3, ERCC4, ERCC5, ERCC6, ERCC8, EXO1, FAN1, FANCA, FANCB, FANCC*) were excluded from the analysis due to a problem with a particular lot of TagSets, which passed initial quality control but was subsequently found to lead to abnormally low NanoString counts. Note that a few of the samples were included in two separate NanoString runs. In these cases, the values from the two runs were averaged in all analyses. The heatmap was produced with the *heatmap.2()* function from the gplots package (version 3.1.1., https://CRAN.R-project.org/package=gplots, accessed on 16 November 2021), using the complete linkage clustering method with 1 – Spearman’s ρ as the distance function. Prior to generating the heatmap, data were scaled so that the mean of each gene was 0 and the standard deviation was 1 so as to normalize for differences in NanoString probe efficiency.

### 2.6. The Cancer Genome Atlas (TCGA) Data Analysis

TCGA ovarian serous cystadenocarcinoma mRNAseq RSEM values were retrieved from http://firebrowse.org/ (last accessed: 22 November 2019). The data were filtered to remove genes with very low read counts using the *filterByExpr()* function from the edgeR package v3.28.1 [24]. The edgeR *calcNormFactors()* function was used to calculate library size normalization factors, which were then used when converting the data into log 2 Counts Per Million (log2CPM) using *voom()*. All edgeR functions were run with default parameter settings. In total, 164 of the 168 genes that were used from the NanoString panel were available in TCGA data (after read count filtering).

## 3. Results

### 3.1. Study Cohort

Our study cohort comprised 42 FFPE tissue samples from patients diagnosed with ovarian cancer, the majority of which were classified as high-grade serous carcinomas (Supplementary Table S2). All tumor tissues had been previously screened for the presence of mutations in the *BRCA1* and *BRCA2* genes, and the results are indicated in Supplementary Table S2. Because most ovarian cancers are probably derived from the fallopian tube epithelium [25,26], we included 18 samples of normal fallopian tubes surgically resected from individuals with no cancer diagnosis in our study.

### 3.2. Quantification of BRCA1 and BRCA2 mRNAs with the nCounter Assay

We used the nCounter assay from NanoString Technologies because this is considered an ideal method for expression profiling in FFPE tissues. The assay counts individual mRNA molecules with a sensitivity similar to real-time PCR but without the bias introduced by enzymatic reactions [27]. This is particularly relevant when using RNA isolated from FFPE specimens because fixation with formaldehyde induces the formation of methylene crosslinks that limit successful reverse transcription and PCR amplification [28]. Moreover, RNA becomes progressively more fragmented in FFPE specimens, particularly when blocks are stored at room temperature [29].

In our study, RNA integrity (RIN) values ranged from 1.8 to 5.0 and concentrations from 19.7 to 353.4 ng/µL (Supplementary Figure S1A and Supplementary Table S2). One of the quality control requirements for the nCounter gene expression assay is that at least 50% of the purified RNA molecules are longer than 300 nucleotides (Supplementary Figure S1B). As shown in Supplementary Figure S1C, the RNA extracted from some samples did not meet this quality requirement and was therefore discarded from further analysis. To assess reproducibility, RNA purified from normal fallopian tubes and tumor samples was repeatedly run with different reagent and probe batches and the results showed similar RNA counts (Supplementary Figure S2).

**Figure 1:**
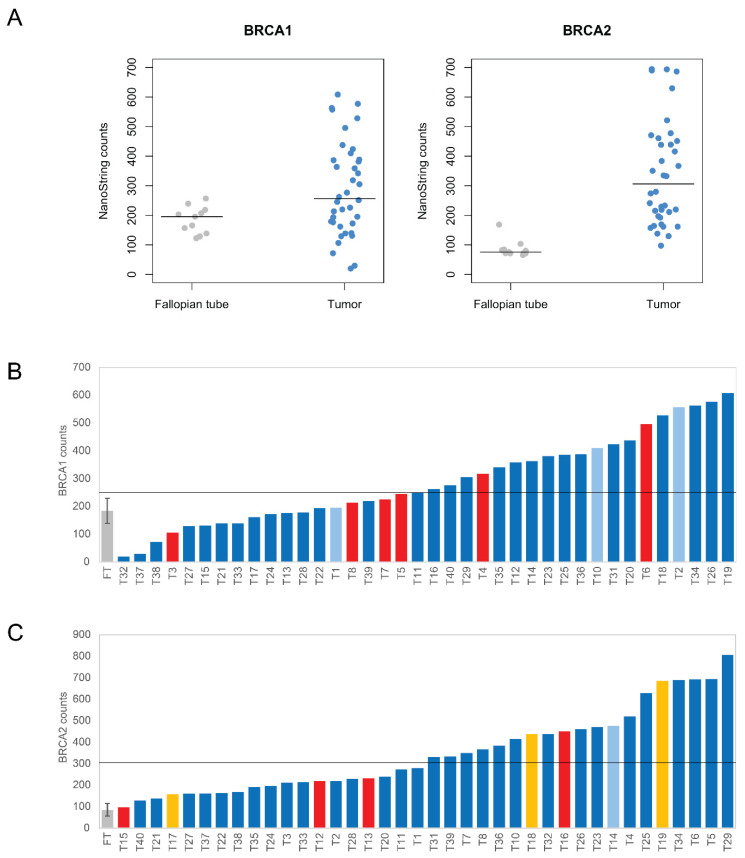
Expression level of *BRCA1* and *BRCA2* genes in 11 normal fallopian tubes and 38 tumor samples analyzed with the NanoString nCounter assay. Tumor samples showed both higher median expression levels as well as greater variability. (**A**) NanoString counts for *BRCA1* and *BRCA2*, in both fallopian tube and tumor samples. The horizontal bar marks the median. (**B**,**C**) Individual mRNA counts for each tumor (T) are shown ordered from lowest to highest *BRCA1* (**B**) and *BRCA2* (**C**) expression levels. Tumors with identified mutations are highlighted (red, frameshift mutations; yellow, Portuguese *BRCA2* founder mutation; light blue, missense mutations). For the 11 normal fallopian tube (FT) samples, the mean and standard deviation are depicted.

To profile the expression of *BRCA1* and *BRCA2* genes, we used the nCounter Vantage™ RNA Panel for DNA Damage and Repair that hybridizes to mRNA from a total of 192 genes, including 12 housekeeping genes and 180 unique genes related to DNA damage and repair pathways (Supplementary Table S3). In normal fallopian tubes, we estimated means of *BRCA1* and *BRCA2* mRNA levels corresponding to 185 and 86 NanoString counts, respectively, with standard deviations of 45 and 29 (Figure 1A). In tumors, the mean level of *BRCA1* and *BRCA2* mRNA corresponded to 280 and 338 NanoString counts, respectively, with standard deviations of 159 and 192. Thus, in contrast to the little variation in mRNA levels of normal fallopian tubes, *BRCA1* and *BRCA2* expression was highly variable among tumors. Some tumors had values comparable to normal tissue while others were up to 10-fold higher than the median of normal samples (Figure 1A–C). The median expression was significantly higher in tumors than in normal tissue for both *BRCA1* (fallopian tube median: 195.23; tumor median: 256.515; *p*~0.010; two-tailed Welch’s *t*-test on ranks) and *BRCA2* (fallopian tube median: 74.13; tumor median: 305.87; *p*~5.59 × 10^−12^; two-tailed Welch’s *t*-test on ranks).

Next, we focused on tumors harboring mutations in the *BRCA1/2* genes (Figure 1B,C). Ten tumors had frameshift mutations located in either *BRCA1* exon 11 or *BRCA2* exons 10 and 11 (Supplementary Figure S3). These mutations generate PTCs and are predicted to elicit NMD according to recently validated rules [30]. Indeed, the PTCs were more than 50–54 bp upstream of the last exon-exon junction, both *BRCA1* and *BRCA2* genes comprised more than two exons, and the PTCs were more than 200 bp downstream of the start codon (Supplementary Figure S3). Despite inter-sample variability, tumors with frameshift mutations were among those with lower expression levels (Figure 1B,C). In these samples, the median *BRCA1* expression was 235.63 (median of all tumors: 256.52), and the median *BRCA2* expression was 225.71 (median of all tumors: 305.87). Three tumors had the c.156–157 insAlu *BRCA2* mutation, which has only been reported in families of Portuguese ancestry. This Portuguese *BRCA2* founder mutation is present in ~30% of all Portuguese families with hereditary breast and ovarian cancer, representing 55% of all *BRCA2* germinal mutation carriers in Portugal [31]. This mutation leads to full in-frame skipping of exon 3 [31,32], which encodes amino acids 23 to 105. Possibly, this protein deletion reduces the interaction between *BRCA2* and *PALB2*, and impairs homologous recombination, thus conferring hypersensitivity to DNA damage [33]. The levels of *BRCA2* expression in these tumors ranged from 157.44 to 686.05, with a median of 438.28 (Figure 1C). Four tumors had missense mutations. In tumors with *BRCA1* missense mutations, the levels of *BRCA1* expression ranged from 195.1 to 557.5, whereas the range of *BRCA2* expression in tumors with frameshifts in *BRCA2* ranged from 273.93 to 477.36 (Figure 1B,C).

### 3.3. Expression Profiling of DNA Damage and Repair Genes

**Figure 2:**
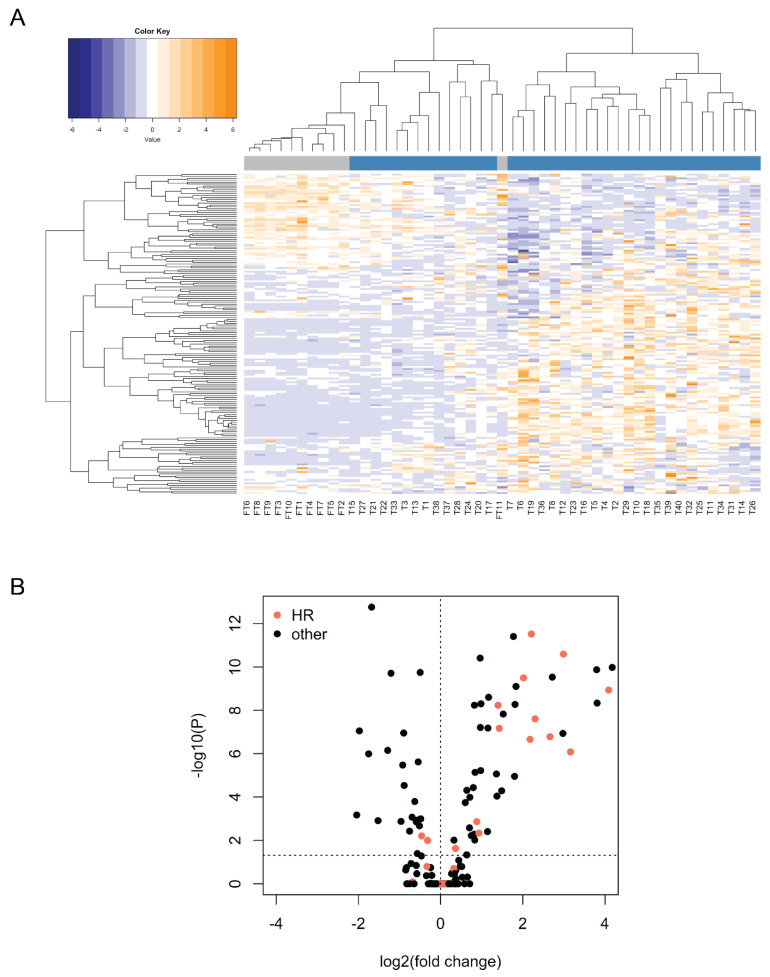
Expression profile of DNA damage and repair genes in 11 normal fallopian tube and 38 tumor samples analyzed with the NanoString nCounter assay. (**A**) Heatmap for mRNA counts obtained with the NanoString nCounter assay for DNA damage and repair genes in 11 normal fallopian tube and 38 tumor samples. Genes are depicted in rows and samples in columns. Both rows and columns have been hierarchically clustered based on Spearman correlations. Normal fallopian tube (FT) samples are labeled in grey, whereas tumor samples (T) are labeled in blue. Note that the data have been scaled and centered in rows (setting the row mean to 0 and the row standard deviation to 1) in order to normalize for differences in probe efficiencies. Tumor and fallopian tube samples cluster separately, with markedly different expression patterns. (**B**) Differential expression of DNA damage repair genes between tumor and fallopian tube samples, as determined through a two-tailed Welch’s *t*-test on ranks with Bonferroni correction performed on the NanoString counts. Log2 fold changes above 0 indicate higher expression in tumors, whereas those below 0 indicate higher expression in fallopian tubes. Most differentially expressed genes show higher expression in tumors.

Having shown that the levels of *BRCA1/2* mRNAs were highly variable between tumor samples, we next analyzed the expression of the additional genes probed with the nCounter Vantage™ RNA Panel for DNA Damage and Repair (Supplementary Table S3). We observed strikingly different expression patterns between normal tissue and many of the tumor samples, although there was high variation between tumors (Figure 2A, see Supplementary Table S4 for the NanoString counts of all the genes in all the samples). We found 72 genes to be differentially expressed between tumor and normal samples (two-tailed Welch’s *t*-test on ranks with Bonferroni correction; genes were considered to be significantly differentially expressed if the corrected *p*-value was below 0.05; Figure 2B, Table 1). Among the 47 genes showing significantly higher expression in tumors, 13 (~28%) belonged to the Homologous Recombination (HR) pathway ((A) in Table 1).

**Figure 3:**
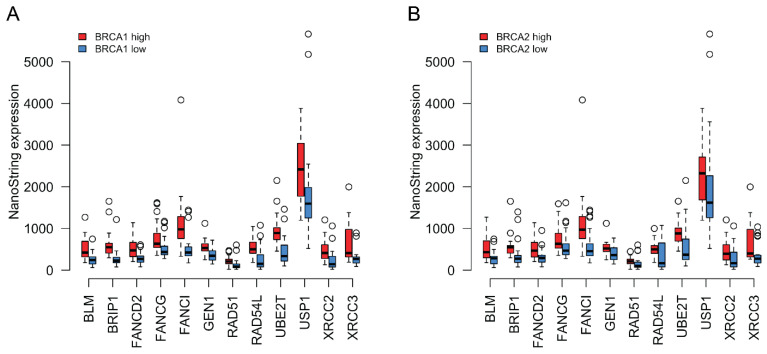
(**A**) Coordinated expression of *BRCA1* with other HR genes that were found upregulated in tumors compared to normal tissue. Red boxplots correspond to tumors where *BRCA1* expression was equal to or above the median for all tumor samples, whereas blue boxplots represent samples where *BRCA1* expression was below the tumor median. The thick horizontal lines show the median. Tumors with higher *BRCA1* expression also tended to show higher expression of the other HR genes depicted. (**B**) Similar to (**A**) but classing the tumors based on *BRCA2* expression instead.

We next asked whether the expression of *BRCA1* and *BRCA2* correlates with the expression of the other HR genes that we found upregulated in tumors. We divided the cohort into low and high groups based on their *BRCA1/2* expression. The low group included tumors with *BRCA1/2* mRNA levels equal to or below the median value, while the high group included tumors with mRNA levels above the median. As shown in Figure 3A,B, the expression of significantly differentially expressed HR genes was systematically downregulated in the *BRCA1/2* low groups and upregulated in the *BRCA1/2* high groups (Supplementary Table S5 contains the Spearman coefficients for the correlations between the NanoStrings’ expression levels of *BRCA1*/*BRCA2* and the genes shown in Figure 3A,B).

HR genes were not the only genes to be upregulated in tumors ((A) in Table 1). It is possible that BRCA expression is not correlated specifically with the expression of other HR genes but rather that all genes that we detected to be upregulated in tumors show similarly correlated expression with each other, independently of the DNA damage pathway that they belong to. To distinguish between these possibilities, we calculated the Spearman correlation coefficient between *BRCA1/2* and each of the non-BRCA HR genes that were upregulated in tumors. We took the mean of these coefficients as our statistic for the extent of co-regulation between *BRCA1/2* and the other upregulated HR genes (*BRCA1*: ~0.59; *BRCA2*: ~0.69; note that a Fisher’s Z-transformation was applied to the correlation coefficients prior to calculating the mean, with the mean of z’ values then converted back to a Spearman’s correlation coefficient). We then performed a simulation where, over 10,000 iterations, we randomly picked 12 of the upregulated genes (12 is the number of upregulated HR genes, excluding *BRCA2*) and calculated the mean correlation coefficient with *BRCA1/2* for this random sample. The mean of the values obtained with the different random samples is the random expectation for how strongly *BRCA1/2* expression is expected to correlate with a random set of upregulated genes (*BRCA1*: ~0.50; *BRCA2*: ~0.53). For both *BRCA1* and *BRCA2*, this random expectation was lower than the value actually observed (*BRCA1*: *p*~0.012; *BRCA2*: ~4.000 × 10^−4^), suggesting that *BRCA1/2* are specifically coregulated with a subset of other HR genes (see Methods for further details on the calculation of empirical *p*-values).

### 3.4. Coordinated Expression of BRCA1/2 and a Subset of HR Genes in TCGA Data

**Figure 4:**
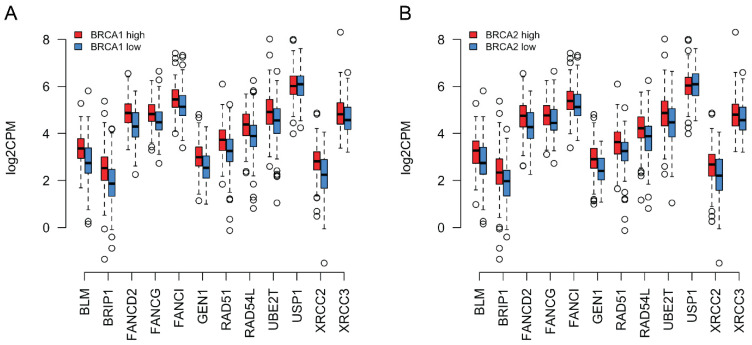
(**A**) RNA-seq expression values of a subset of HR genes (see legend to Figure 3) in log2 Counts Per Million (log2CPM) for ovarian cancer samples from the TCGA database. Median *BRCA1* expression was calculated for the TCGA ovarian cancer samples. Samples where *BRCA1* expression was equal to or above this median are depicted in red, whereas those where it was below this median are depicted in blue. The thick horizontal lines show the median. Again, the tumors with higher *BRCA1* expression also tended to show higher expression of the other depicted genes. (**B**) Similar to (**A**) but classing the tumors based on *BRCA2* expression instead.

In order to determine whether the coordinated expression of *BRCA1/2* and the 12 other HR genes identified above could be confirmed in an independent data set, we obtained mRNA sequencing data for genes related to DNA damage and repair pathways in 299 ovarian cancer samples from The Cancer Genome Atlas (TCGA). Similar to our analysis of NanoString data, we divided the samples into a high and low *BRCA1/2* expression group along the median. We asked whether the HR genes found to be upregulated in tumors in NanoString data showed evidence of co-regulation with *BRCA1/2* also in TCGA data. As can be observed in Figure 4A,B, when *BRCA1/2* was low, the expression of HR genes was also low compared to their expression when *BRCA1/2* was high (see Supplementary Table S6 for the Spearman coefficients for the correlations between the expression levels of *BRCA1*/*BRCA2* and the genes shown in Figure 4A,B). TCGA data thus support co-regulation of *BRCA1/2* with the other HR genes. To know if this effect was significantly stronger for HR genes than for upregulated genes in general, we also repeated the simulation reported in the previous section with TCGA data (using the list of significantly upregulated genes that was determined with NanoString above). We obtained a true mean Spearman correlation coefficient of ~0.30 (random expectation: ~0.22; empirical *p*~0.028) for *BRCA1,* and ~0.41 for *BRCA2* (random expectation: ~0.28; empirical *p*~0.001).

### 3.5. Targeted Quantitation of BRCA1/2 mRNA by ddPCR

**Figure 5:**
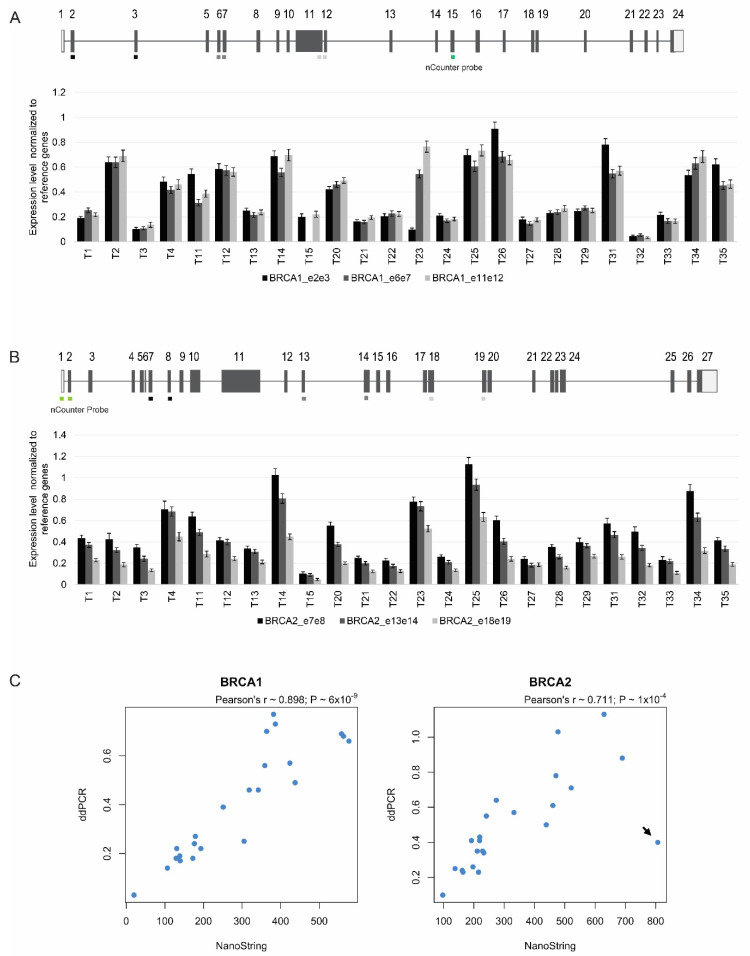
ddPCR quantitation of *BRCA1* and *BRCA2* expression in 24 ovarian cancers. (**A**,**B**) The normalized ratio of *BRCA1* (**A**) and *BRCA2* (**B**) mRNA to the three reference genes is plotted. Error bars represent 95% confidence intervals of the measurements. The different regions targeted by the ddPCR assays are indicated (exons 2/3, 6/7, and 11/12 of *BRCA1*; exons 7/8, 13/14, and 18/19 of *BRCA2*). The region targeted by the nCounter assay in each gene is denoted in green. (**C**) A strong correlation was observed between *BRCA1* and *BRCA2* expression values estimated with the NanoString nCounter and ddPCR assays. Arrow denotes tumor sample with discordant values, with a high *BRCA2* mRNA value estimated by the NanoString nCounter system but a low value quantified by ddPCR.

Having established that *BRCA1* and *BRCA2* expression was correlated with that of other HR genes in ovarian cancers, we sought to develop a targeted and less costly approach to measure *BRCA1/2* mRNA levels in FFPE clinical samples. We decided to explore droplet digital PCR (ddPCR) because this approach was previously shown to provide reliable quantification of *HER2* mRNA in FFPE breast cancer specimens [34,35]. We further opted for a one-step method in which the cDNA synthesis is initiated with the reverse primer within each droplet, thus minimizing bias introduced by retro transcription efficiency. We designed primers and probes that amplified and detected different regions of the *BRCA1* and *BRCA2* transcripts (Figure 5A,B). As reference genes, we analyzed *GUSB, POLR1E,* and *NUBP1*. Duplex experiments were performed targeting simultaneously either *BRCA1*, *BRCA2*, *POLR1E* or *NUBP1* and *GUSB* (Supplementary Figure S4). The number of read droplets varied from 5549 to 19,264. Values obtained in control experiments with no retro transcriptase were null for 75% of the cases, with a maximum of 0.16 copies/µL. The concentration of *BRCA1/2* target sequences varied from 1.8 to 57.2 copies/µL. This yield was approximately 15 times less than the CPD values obtained from non-fixed, freshly isolated PBMCs.

We tested 24 tumor samples for *BRCA1* and *BRCA2* mRNA levels, normalized to the reference genes *GUSB*, *POLR1E,* and *NUBP1*. For each tumor, we performed three different assays for *BRCA1*, and another three distinct assays for *BRCA2* (Figure 5A,B). Similar values were observed with the assays targeting the different regions of the same mRNA molecule in PBMCs from 10 individuals, as well as in a sub-set of the tumor samples; however, in other tumor samples, the levels differed among the three regions analyzed (Figure 5A,B). For further analysis (see below), we selected the assay that consistently revealed higher mRNA levels across all tumors (i.e., for *BRCA1* the assay that targets exons 11/12, and for *BRCA2* the assay that targets exons 7/8).

A comparison between the relative expression values for *BRCA1* and *BRCA2* estimated by the NanoString nCounter system and ddPCR showed a significant correlation (Figure 5C); only one tumor sample was clearly discordant, with a high *BRCA2* mRNA value estimated by the NanoString nCounter system but a low value quantified by ddPCR (Figure 5C, arrow). These results indicate that ddPCR is a reliable method to quantitate levels of *BRCA1* and *BRCA2* mRNA in clinical ovarian cancer samples.

## 4. Discussion

In this study, we measured the levels of *BRCA1* and *BRCA2* mRNA in ovarian cancers and normal fallopian tubes, the organ from which most ovarian cancers originate [25,26]. We used the nCounter assay from NanoString Technologies to circumvent the limitations caused by varying extents of RNA degradation in FFPE samples. We found that *BRCA1* and *BRCA2* expression was more variable among tumors than across normal tissues, with many tumors showing higher mRNA values compared to normal fallopian tubes. Previous studies showed that the expression of *BRCA1* and *BRCA2* genes is upregulated in rapidly proliferating cells [36,37]. Thus, the higher level of *BRCA1* and *BRCA2* mRNA observed in tumors may result from the proliferation status of cancer cells. Consistent with the view that the cellular proliferation rate is higher in ovarian cancers than in normal fallopian tubes, we identified several cell-cycle-related genes that were significantly upregulated in the tumors ((A) in Table 1). These included genes encoding proteins that stimulate cell proliferation, such as KRAS and AURKA, the mitotic checkpoint proteins BUB1B and MAD2L2, as well as proteins involved in DNA replication and repair of replication-associated lesions, such as Replication factor C subunit 4 (RFC4), DNA replication helicase/nuclease 2 (DNA2), DNA polymerase delta 1 (POLD1), DNA polymerase delta 4, accessory subunit (POLD4), DNA polymerase epsilon subunit 2 (POLE2), Flap structure-specific endonuclease 1 (FEN1), and Proliferating cell nuclear antigen (PCNA).

All tumors expressed *BRCA2* mRNA at levels similar to or higher than the median of normal tissues (Figure 1). In contrast, a subset of tumors expressed *BRCA1* mRNA at lower levels than the median of normal tissues (Figure 1). A possible cause for such a reduction could be epigenetic silencing via promoter hypermethylation, which has been detected in the *BRCA1* gene in 10–20% of ovarian cancers [13,38,39]. Additional mechanisms that can lead to reduced *BRCA1/2* expression include microRNA-mediated regulation [40] and frameshift-inducing mutations that trigger mRNA degradation by NMD. Notably, the majority of known germline and somatic *BRCA1/2* mutations are frameshift insertions or deletions [13], and in our study, among tumors with lower levels of expression, we identified eight cases harboring frameshift mutations that are predicted to activate NMD. The frameshift mutations that we identified in the *BRCA1/2* genes were heterozygous. Thus, only expression of the mutant allele is expected to be reduced. However, high variability of mRNA levels was observed between different tumors with frameshift mutations. One possibility is that transcription may be differentially upregulated depending on the proliferation rate of tumor cells. Alternatively, the normal allele may be differentially inactivated by loss of heterozygosity, which has been reported in a large fraction of ovarian cancers [13].

Several lines of evidence suggest that reduced *BRCA1/2* gene expression in tumors leads to defective DNA repair via HR. Indeed, downregulation of BRCA1 protein induced by overexpression of miR-182 was sufficient to impair HR-mediated repair and hypersensitize breast tumor cells to PARPi [40]. PI3K inhibition was also shown to downregulate *BRCA1/2* expression leading to DNA damage and sensitization to PARPi [41]. Phosphatidyl-inositol-3-kinases (PI3Ks) constitute a family of lipid kinases that when activated trigger a signaling cascade. One of the key proteins involved in this pathway is AKT/protein kinase B [42]. BRCA1 is one of many targets phosphorylated by AKT [43]. Genomic alterations leading to activation of the PI3K pathway are present in ovarian cancer [13], and both preclinical and clinical studies suggest that PI3K pathway inhibitors improve the cancer response to PARP inhibition [44]. Moreover, knockdown of *BRCA1* in MCF-10A cells, which are immortal human mammary epithelial cells of nonmalignant origin, caused HR deficiency without affecting cell cycle progression and resulted in downregulation of mRNAs encoding several other HR factors and enzymes required for double-strand break repair [18]. This study further revealed that knockdown of distinct HR genes resulted in similar transcriptomic changes, suggesting that the expression of HR genes is coordinately regulated in HR deficient cells [18]. Our work extends these observations by showing that the expression of *BRCA1* and *BRCA2* genes significantly correlates with that of an additional 12 HR genes in clinical ovarian cancer samples. This implies that reduced levels of *BRCA1/2* mRNA in tumors may result from a variety of genetic or epigenetic alterations in either the *BRCA1/2* genes or other HR genes.

We further found an excellent correlation between *BRCA1/2* gene expression levels in tumors analyzed by the NanoString nCounter system and ddPCR. Thus, our study demonstrates, for the first time, that ddPCR can be used for the accurate quantitation of *BRCA1* and *BRCA2* mRNA in FFPE ovarian cancer specimens. Compared to traditional RT-PCR approaches applied to clinical samples, ddPCR offers greater precision, improved reproducibility, and superior diagnostic performance [45]. In the particular case of RNA extracted from FFPE samples, ddPCR has the advantage of allowing extremely low target quantitation [46].

Previous studies described an association between reduced BRCA1 mRNA or protein levels and responsiveness to cisplatin therapy in different types of cancer [47,48,49,50,51,52,53]. Furthermore, platinum sensitivity correlated with lower BRCA1/2 mRNA expression in ovarian cancers [54]. Thus, measuring the levels of *BRCA1* and *BRCA2* mRNAs in ovarian cancers using either the NanoString nCounter system or ddPCR may help predict which patients will benefit the most from platinum-based chemotherapy and PARPi when DNA tests are negative or inconclusive. Future validation studies in large cohorts are needed to define cut-off values of *BRCA1/2* mRNA levels capable of identifying which tumors are more sensitive to PARPi.

## Figures and Tables

**Table 1:** Genes significantly differentially expressed in tumors.

**(A) Genes Upregulated in Tumors.**	
**Pathway**	**Genes**
Apoptosis	BCL2L1, CASP8, MYD88
Base excision repair	APEX2, FEN1, NEIL3, PARP1, SMUG1, UNG
Base excision repair—Translesion Synthesis—Cell Cycle and Signaling	PCNA
Cell Cycle and Signaling	AURKA, BUB1B, CDKN2A, KRAS, RAD21, RM12, SUMO3
Checkpoint Activation	H2AFX
Checkpoint Activation—Cell Cycle and Signaling	CHEK1/2
Homologous Recombination and Fanconi Anemia	BRIP1, BLM, BRCA2, FANCD2, FANCG, FANCI, GEN1, RAD51, RAD54L, UBE2T, USP1, XRCC2, XRCC3
Independent Repair Enzymes/Polymerases	DNA2, POLQ
Independent Repair Enzymes/Polymerases–Base excision repair	POLD1, POLD4, POLE2
Independent Repair Enzymes/Polymerases—Cell Cycle and Signaling	MAD2L2
Independent Repair Enzymes/Polymerases–Nucleotide Excision Repair	POLR2D, POLR2H
Independent Repair Enzymes/Polymerases–Translesion Synthesis	HLTF
Mismatch Repair	MSH2
Mismatch Repair–Translesion Synthesis	RFC4
Non-homologous End Joining—Cell Cycle and Signaling	PRKDC
Translesion Synthesis	RAD18
Housekeeping	SF3FA3
**(B) Genes downregulated in tumors.**	
**Pathway**	**Genes**
Apoptosis	AKT3, BCL2, NFKB2, PIK3R1
Base Excision Repair	NEIL1, NEIL2, OGG1
Cell Cycle and Signaling	ABL1, CCND2, CDKN1A, EGFR
Checkpoint Activation	TIPIN
Homologous Recombination and Fanconi Anemia	RAD51B, WRN
Independent Repair Enzymes/Polymerases	ALKBH3, CRY1, POLK, REV1
Independent Repair Enzymes/Polymerases–Translesion Synthesis	POLI
Mismatch Repair	MSH3
Non-homologous End Joining	LIG4
Nucleotide Excision Repair	XPA
Nucleotide Excision Repair—Apoptosis	PTEN
Housekeeping	COG7, NUBP1

## Data Availability

The data presented in this study are available in Supplementary Table S4.

## Supplementary Materials

The following are available online at https://www.mdpi.com/article/10.3390/biomedicines10020199/s1, Figure S1: FFPE Samples for NanoString analysis. (**A**) Evaluation of RNA quantity and quality with the 4200 TapeStation (Agilent Technologies, Santa Clara, CA) using the Agilent RNA ScreenTape Assay. Concentration (ng/μL) and RIN (RNA integrity number) values for a representative group of control and tumor samples are shown. (**B**) Representative electropherograms of RNA retrieved from fallopian tube sample FT1 and ovarian tumor sample T29. The percent of samples greater than 300 nt was estimated by the percent of the sample between 50–300 nt and subtracting that quantity from 100%. (**C**) Overview of FFPE samples, Figure S2: NanoString expression counts for DNA damage repair genes, in samples that were analyzed in two different runs using different reagent batches, Figure S3: Schematic representation of the *BRCA1* (**A**) and *BRCA2* (**B**) genes with the locations of the frameshift mutations analyzed (indicated with red dots), Figure S4: Example of ddPCR data output (Tumor 11). One-dimensional scatter plots showing duplex experiments targeting different *BRCA1* and *BRCA2* regions detected by FAM fluorescence (**A**) and *GUSB* detected with HEX fluorescence (**B**). Positive droplets are depicted in blue (**A**) and green (**B**); negative droplets are depicted in grey (**A**,**B**); the threshold line is depicted in pink (**A**,**B**). The plots shown in panel **A** correspond to the following assays: *BRCA1* exons 2/3 (A01), 6/7 (A02), 11/12 (A03), and *BRCA2* exons 7/8 (A04), 13/14 (A05), and 18/19 (A06), Table S1: Primers and probes used for droplet digital PCR analysis, Table S2: Information on the samples. (**A**) Tumor samples used in Nanostring analysis. (**B**) Tumor samples excluded from the Nanostring analysis due to low RNA concentration. (**C**) Fallopian tube samples. RNA integrity (RIN) values and RNA concentrations (ng/µL) are indicated, Table S3: List of genes in nCounter Vantage™ RNA Panel for DNA Damage and Repair and signature descriptions. This panel includes 192 genes of which 180 are unique genes related to DNA damage and repair pathways and 12 are housekeeping genes used for data normalization, Table S4: Normalized NanoString counts of all the genes in all the samples, Table S5: Spearman coefficients for the correlations between the NanoStrings’ expression levels of *BRCA1*/*BRCA2* and the genes shown in Figure 3A,B, Table S6: Spearman coefficients for the correlations between the expression levels of *BRCA1*/*BRCA2* and the genes shown in Figure 4A,B.

## References

[1] Torre L.A., Trabert B., DeSantis C.E., Miller K.D., Samimi G., Runowicz C.D., Gaudet M.M., Jemal A., Siegel R.L. (2018). Ovarian cancer statistics, 2018. CA Cancer J. Clin..

[2] Howlader N., Noone A.M., Krapcho M., Miller D., Brest A., Yu M., Ruhl J., Tatalovich Z., Mariotto A., Lewis D.R. (2019). SEER Cancer Statistics Review, 1975–2016.

[3] Lheureux S., Gourley C., Vergote I., Oza A.M. (2019). Epithelial ovarian cancer. Lancet.

[4] Cortez A.J., Tudrej P., Kujawa K.A., Lisowska K.M. (2018). Advances in ovarian cancer therapy. Cancer Chemother. Pharmacol..

[5] Mateo J., Lord C.J., Serra V., Tutt A., Balmaña J., Castroviejo-Bermejo M., Cruz C., Oaknin A., Kaye S.B., de Bono J.S. (2019). A decade of clinical development of PARP inhibitors in perspective. Ann. Oncol. Off. J. Eur. Soc. Med. Oncol..

[6] Rose M., Burgess J.T., O’Byrne K., Richard D.J., Bolderson E. (2020). PARP Inhibitors: Clinical Relevance, Mechanisms of Action and Tumor Resistance. Front. Cell Dev. Biol..

[7] Ray Chaudhuri A., Nussenzweig A. (2017). The multifaceted roles of PARP1 in DNA repair and chromatin remodelling. Nat. Rev. Mol. Cell Biol..

[8] Farmer H., McCabe N., Lord C.J., Tutt A.N., Johnson D.A., Richardson T.B., Santarosa M., Dillon K.J., Hickson I., Knights C. (2005). Targeting the DNA repair defect in BRCA mutant cells as a therapeutic strategy. Nature.

[9] Bryant H.E., Schultz N., Thomas H.D., Parker K.M., Flower D., Lopez E., Kyle S., Meuth M., Curtin N.J., Helleday T. (2005). Specific killing of BRCA2-deficient tumours with inhibitors of poly(ADP-ribose) polymerase. Nature.

[10] Li X., Heyer W.D. (2008). Homologous recombination in DNA repair and DNA damage tolerance. Cell Res..

[11] Wright W.D., Shah S.S., Heyer W.D. (2018). Homologous recombination and the repair of DNA double-strand breaks. J. Biol. Chem..

[12] Gelmon K.A., Tischkowitz M., Mackay H., Swenerton K., Robidoux A., Tonkin K., Hirte H., Huntsman D., Clemons M., Gilks B. (2011). Olaparib in patients with recurrent high-grade serous or poorly differentiated ovarian carcinoma or triple-negative breast cancer: A phase 2, multicentre, open-label, non-randomised study. Lancet. Oncol..

[13] Network C.G.A.R. (2011). Integrated genomic analyses of ovarian carcinoma. Nature.

[14] Fong P.C., Yap T.A., Boss D.S., Carden C.P., Mergui-Roelvink M., Gourley C., De Greve J., Lubinski J., Shanley S., Messiou C. (2010). Poly(ADP)-ribose polymerase inhibition: Frequent durable responses in BRCA carrier ovarian cancer correlating with platinum-free interval. J. Clin. Oncol. Off. J. Am. Soc. Clin. Oncol..

[15] Moschetta M., George A., Kaye S.B., Banerjee S. (2016). BRCA somatic mutations and epigenetic BRCA modifications in serous ovarian cancer. Ann. Oncol. Off. J. Eur. Soc. Med. Oncol..

[16] Roy R., Chun J., Powell S.N. (2011). BRCA1 and BRCA2: Different roles in a common pathway of genome protection. Nat. Rev. Cancer.

[17] Vaz-Drago R., Custódio N., Carmo-Fonseca M. (2017). Deep intronic mutations and human disease. Hum. Genet..

[18] Peng G., Chun-Jen Lin C., Mo W., Dai H., Park Y.Y., Kim S.M., Peng Y., Mo Q., Siwko S., Hu R. (2014). Genome-wide transcriptome profiling of homologous recombination DNA repair. Nat. Commun..

[19] Chang Y.F., Imam J.S., Wilkinson M.F. (2007). The nonsense-mediated decay RNA surveillance pathway. Annu. Rev. Biochem..

[20] Hu Z., Yau C., Ahmed A.A. (2017). A pan-cancer genome-wide analysis reveals tumour dependencies by induction of nonsense-mediated decay. Nat. Commun..

[21] Taylor S.C., Nadeau K., Abbasi M., Lachance C., Nguyen M., Fenrich J. (2019). The Ultimate qPCR Experiment: Producing Publication Quality, Reproducible Data the First Time. Trends Biotechnol..

[22] Huggett J.F., Foy C.A., Benes V., Emslie K., Garson J.A., Haynes R., Hellemans J., Kubista M., Mueller R.D., Nolan T. (2013). The digital MIQE guidelines: Minimum Information for Publication of Quantitative Digital PCR Experiments. Clin. Chem..

[23] North B.V., Curtis D., Sham P.C. (2003). A note on calculation of empirical P values from Monte Carlo procedure. Am. J. Hum. Genet..

[24] Robinson M.D., McCarthy D.J., Smyth G.K. (2010). edgeR: A Bioconductor package for differential expression analysis of digital gene expression data. Bioinformatics.

[25] Labidi-Galy S.I., Papp E., Hallberg D., Niknafs N., Adleff V., Noe M., Bhattacharya R., Novak M., Jones S., Phallen J. (2017). High grade serous ovarian carcinomas originate in the fallopian tube. Nat. Commun..

[26] Hu Z., Artibani M., Alsaadi A., Wietek N., Morotti M., Shi T., Zhong Z., Santana Gonzalez L., El-Sahhar S., KaramiNejadRanjbar M. (2020). The Repertoire of Serous Ovarian Cancer Non-genetic Heterogeneity Revealed by Single-Cell Sequencing of Normal Fallopian Tube Epithelial Cells. Cancer Cell.

[27] Geiss G.K., Bumgarner R.E., Birditt B., Dahl T., Dowidar N., Dunaway D.L., Fell H.P., Ferree S., George R.D., Grogan T. (2008). Direct multiplexed measurement of gene expression with color-coded probe pairs. Nat. Biotechnol..

[28] Masuda N., Ohnishi T., Kawamoto S., Monden M., Okubo K. (1999). Analysis of chemical modification of RNA from formalin-fixed samples and optimization of molecular biology applications for such samples. Nucleic Acids Res..

[29] von Ahlfen S., Missel A., Bendrat K., Schlumpberger M. (2007). Determinants of RNA quality from FFPE samples. PLoS ONE.

[30] Lindeboom R.G., Supek F., Lehner B. (2016). The rules and impact of nonsense-mediated mRNA decay in human cancers. Nat. Genet..

[31] Peixoto A., Santos C., Pinto P., Pinheiro M., Rocha P., Pinto C., Bizarro S., Veiga I., Principe A.S., Maia S. (2015). The role of targeted BRCA1/BRCA2 mutation analysis in hereditary breast/ovarian cancer families of Portuguese ancestry. Clin. Genet..

[32] Machado P.M., Brandão R.D., Cavaco B.M., Eugénio J., Bento S., Nave M., Rodrigues P., Fernandes A., Vaz F. (2007). Screening for a BRCA2 rearrangement in high-risk breast/ovarian cancer families: Evidence for a founder effect and analysis of the associated phenotypes. J. Clin. Oncol. Off. J. Am. Soc. Clin. Oncol..

[33] Caputo S.M., Léone M., Damiola F., Ehlen A., Carreira A., Gaidrat P., Martins A., Brandão R.D., Peixoto A., Vega A. (2018). Full in-frame exon 3 skipping of BRCA2 confers high risk of breast and/or ovarian cancer. Oncotarget.

[34] Heredia N.J., Belgrader P., Wang S., Koehler R., Regan J., Cosman A.M., Saxonov S., Hindson B., Tanner S.C., Brown A.S. (2013). Droplet Digital™ PCR quantitation of HER2 expression in FFPE breast cancer samples. Methods.

[35] Meehan K., Clynick B., Mirzai B., Maslen P., Harvey J.M., Erber W.N. (2017). HER2 mRNA transcript quantitation in breast cancer. Clin. Transl. Oncol. Off. Publ. Fed. Span. Oncol. Soc. Natl. Cancer Inst. Mex..

[36] Gudas J.M., Li T., Nguyen H., Jensen D., Rauscher F.J., Cowan K.H. (1996). Cell cycle regulation of BRCA1 messenger RNA in human breast epithelial cells. Cell Growth Differ. Mol. Biol. J. Am. Assoc. Cancer Res..

[37] Rajan J.V., Marquis S.T., Gardner H.P., Chodosh L.A. (1997). Developmental expression of Brca2 colocalizes with Brca1 and is associated with proliferation and differentiation in multiple tissues. Dev. Biol..

[38] Baldwin R.L., Nemeth E., Tran H., Shvartsman H., Cass I., Narod S., Karlan B.Y. (2000). BRCA1 promoter region hypermethylation in ovarian carcinoma: A population-based study. Cancer Res..

[39] Esteller M., Silva J.M., Dominguez G., Bonilla F., Matias-Guiu X., Lerma E., Bussaglia E., Prat J., Harkes I.C., Repasky E.A. (2000). Promoter hypermethylation and BRCA1 inactivation in sporadic breast and ovarian tumors. J. Natl. Cancer Inst..

[40] Moskwa P., Buffa F.M., Pan Y., Panchakshari R., Gottipati P., Muschel R.J., Beech J., Kulshrestha R., Abdelmohsen K., Weinstock D.M. (2011). miR-182-mediated downregulation of BRCA1 impacts DNA repair and sensitivity to PARP inhibitors. Mol. Cell.

[41] Ibrahim Y.H., García-García C., Serra V., He L., Torres-Lockhart K., Prat A., Anton P., Cozar P., Guzmán M., Grueso J. (2012). PI3K inhibition impairs BRCA1/2 expression and sensitizes BRCA-proficient triple-negative breast cancer to PARP inhibition. Cancer Discov..

[42] Porta C., Paglino C., Mosca A. (2014). Targeting PI3K/Akt/mTOR Signaling in Cancer. Front. Oncol..

[43] Nelson A.C., Lyons T.R., Young C.D., Hansen K.C., Anderson S.M., Holt J.T. (2010). AKT regulates BRCA1 stability in response to hormone signaling. Mol. Cell. Endocrinol..

[44] Dong C., Wu J., Chen Y., Nie J., Chen C. (2021). Activation of PI3K/AKT/mTOR Pathway Causes Drug Resistance in Breast Cancer. Front. Pharmacol..

[45] Hindson C.M., Chevillet J.R., Briggs H.A., Gallichotte E.N., Ruf I.K., Hindson B.J., Vessella R.L., Tewari M. (2013). Absolute quantification by droplet digital PCR versus analog real-time PCR. Nat. Methods.

[46] Taylor S.C., Laperriere G., Germain H. (2017). Droplet Digital PCR versus qPCR for gene expression analysis with low abundant targets: From variable nonsense to publication quality data. Sci. Rep..

[47] Wang L., Wei J., Qian X., Yin H., Zhao Y., Yu L., Wang T., Liu B. (2008). ERCC1 and BRCA1 mRNA expression levels in metastatic malignant effusions is associated with chemosensitivity to cisplatin and/or docetaxel. BMC Cancer.

[48] Quinn J.E., James C.R., Stewart G.E., Mulligan J.M., White P., Chang G.K., Mullan P.B., Johnston P.G., Wilson R.H., Harkin D.P. (2007). BRCA1 mRNA expression levels predict for overall survival in ovarian cancer after chemotherapy. Clin. Cancer Res. Off. J. Am. Assoc. Cancer Res..

[49] Carser J.E., Quinn J.E., Michie C.O., O’Brien E.J., McCluggage W.G., Maxwell P., Lamers E., Lioe T.F., Williams A.R., Kennedy R.D. (2011). BRCA1 is both a prognostic and predictive biomarker of response to chemotherapy in sporadic epithelial ovarian cancer. Gynecol. Oncol..

[50] Gao Y., Zhu J., Zhang X., Wu Q., Jiang S., Liu Y., Hu Z., Liu B., Chen X. (2013). BRCA1 mRNA expression as a predictive and prognostic marker in advanced esophageal squamous cell carcinoma treated with cisplatin- or docetaxel-based chemotherapy/chemoradiotherapy. PLoS ONE.

[51] Huang Y., Wu P., Liu B., Du J. (2016). Successful personalized chemotherapy for metastatic gastric cancer based on quantitative BRCA1 mRNA expression level: A case report. Oncol. Lett..

[52] Rosell R., Perez-Roca L., Sanchez J.J., Cobo M., Moran T., Chaib I., Provencio M., Domine M., Sala M.A., Jimenez U. (2009). Customized treatment in non-small-cell lung cancer based on EGFR mutations and BRCA1 mRNA expression. PLoS ONE.

[53] Taron M., Rosell R., Felip E., Mendez P., Souglakos J., Ronco M.S., Queralt C., Majo J., Sanchez J.M., Sanchez J.J. (2004). BRCA1 mRNA expression levels as an indicator of chemoresistance in lung cancer. Hum. Mol. Genet..

[54] Tsibulak I., Wieser V., Degasper C., Shivalingaiah G., Wenzel S., Sprung S., Lax S.F., Marth C., Fiegl H., Zeimet A.G. (2018). BRCA1 and BRCA2 mRNA-expression prove to be of clinical impact in ovarian cancer. Br. J. Cancer.

