# Supplementary material for: Expression Profiling in Ovarian Cancer Reveals Coordinated Regulation of BRCA1/2 and Homologous Recombination Genes
Source: Biomedicines. 2022 Jan 18;10(2):199. doi: 10.3390/biomedicines10020199 (PMC8868827; doi:10.3390/biomedicines10020199)
Supplement: Supplementary file 1 [file biomedicines-10-00199-s001.zip › Table S1.pdf]

Table S1 - Primers and probes used for droplet digital PCR analysis.

Primer-probe set		oligo name	sequence	size	amplicon
**BRCA1**					
BRCA1_e2e3	forward	BRCA1_E2F	TTGAAGAAGTACAAAATGTCATTAATGC	28	90
	reverse	BRCA1_E3R	TTGTGGAGACAGGTTCCTTG	20	
	hyb probe	BRCA1_E2E3_PBF	AGAGTGTCCCATCTGTCTGGAGTTGA	26	
BRCA1_e6e7	forward	BRCA1_E6F2	ACAGAGTGAACCCGAAAATCC	21	87
	reverse	BRCA1_E7R2	GTCCTCAGAGTTCTCACAGTTC	22	
	hyb probe	BRCA1_E6E7_PBF	CCTTGCAGGAAACCAGTCTCAGTGT	25	
BRCA1_e11e12	forward	BRCA1_E11F	AGAGTGAAACAAGCGTCTCTG	21	89
	reverse	BRCA1_E12R	ATGTTGCATGGTATCCCTCTG	21	
	hyb probe	BRCA1_E11E12_PBF	CTGCTCAGGGCTATCCTCTCAGAGT	25	
**BRCA2**					
BRCA2_e7e8	forward	BRCA2_E7F	CTTGGTCAAGTTCTTTAGCTACAC	24	90
	reverse	BRCA2_E8R	GAGGAAATACAGTTTCAGATGCTTC	25	
	hyb probe	BRCA2_E7E8_PBF	ACCCACCCTTAGTTCTACTGTGCTCA	26	
BRCA2_e13e14	forward	BRCA2_E13F	TTCTTTAGAGCCGATTACCTGTG	23	84
	reverse	BRCA2_E14R	CCAGGTGCGGTAAAATTTGG	20	
	hyb probe	BRCA2_E13E14_PBR	ACGTTCCTTAGTTGTGCGAAAGGGT	25	
BRCA2_e18e19	forward	BRCA2_E18F2	TGAAGCCCCAGAATCTCTTATG	22	89
	reverse	BRCA2_E19R	GGTCAGGAAAGAATCCAAGTTTG	23	
	hyb probe	BRCA2_E18E19_PBR	CGAGCAGGCCGAGTACTGTTAGC	23	
**NUBP1**					
NUBP1_e6e7	forward	NUBP1_E6F	ATCGATGGAGCAGTGATCATC	21	82
	reverse	NUBP1_E7R	CTTGCGGCAGAAGTTGATTTC	21	
	hyb probe	NUBP1_E7_PR	CCGGACATCCTGGAGTGACACC	22	
**POLR1E**					
POLR1E_e9e10	forward	POLR1E_E9F	TCAAATTTCGAGCTCATAGGGTAG	24	78
	reverse	POLR1E_E10R	GTTTGGTGTTGATGATGTGGG	21	
	hyb probe	POLR1E_E9E10_PF	CGGAAAAGTGCTCTGGGACCTGG	23	
**GUSB**					
GUSB_e11e12	forward	GUSB_E11F	GCCGATTTCATGACTGAACAG	21	80
	reverse	GUSB_E12R	TTTGGTTGTCTCTGCCGAG	19	
	hyb probe	GUSB_E12_PBR	TCCCCTTTTTATTCCCCAGCACTCTC	26	
