# Supplementary material for: Expression Profiling in Ovarian Cancer Reveals Coordinated Regulation of BRCA1/2 and Homologous Recombination Genes
Source: Biomedicines. 2022 Jan 18;10(2):199. doi: 10.3390/biomedicines10020199 (PMC8868827; doi:10.3390/biomedicines10020199)
Supplement: Supplementary file 1 [file biomedicines-10-00199-s001.zip › Table S5.pdf]

Table S5: Spearman coefficients for the correlations between the NanoStrings expression levels of *BRCA1/BRCA2* and the genes shown in Figure 3A/B.

**Gene**	**BRCA1**	**BRCA2**
BLM	0.636940584309005	0.748331327278696
BRCA1	1	0.606083816610132
BRCA2	0.606083816610132	1
BRIP1	0.776999671736514	0.725571725571726
FANCD2	0.607396870554765	0.664295874822191
FANCG	0.567348725243462	0.63475216106795
FANCI	0.63912900755006	0.827333406280775
GEN1	0.477185687712003	0.615931721194879
RAD51	0.382636361248409	0.521896290149118
RAD54L	0.6207462523252	0.751832804464383
UBE2T	0.654447970237444	0.755990808622388
USP1	0.307364044206149	0.353102089944195
XRCC2	0.569099463836306	0.67064230222125
XRCC3	0.697997592734435	0.764744501586607
