# Supplementary material for: Expression Profiling in Ovarian Cancer Reveals Coordinated Regulation of BRCA1/2 and Homologous Recombination Genes
Source: Biomedicines. 2022 Jan 18;10(2):199. doi: 10.3390/biomedicines10020199 (PMC8868827; doi:10.3390/biomedicines10020199)
Supplement: Supplementary file 1 [file biomedicines-10-00199-s001.zip › Table S6.pdf]

Table S6: Spearman coefficients for the correlations between the expression levels of *BRCA1/BRCA2* and the genes shown in Figure 4A/B.

**Gene**	**BRCA1**	**BRCA2**
BLM	0.368589706179435	0.532198154923571
BRCA1	1	0.285449035936343
BRCA2	0.285449035936343	1
BRIP1	0.412397477048776	0.432161343179727
FANCD2	0.403458957150232	0.545295952952796
FANCG	0.293784202374806	0.276682004893268
FANCI	0.23530291127023	0.406628358510471
GEN1	0.390609413930103	0.505119077012862
RAD51	0.318195775627932	0.344412920024242
RAD54L	0.325662723620121	0.436083589593949
UBE2T	0.242807119929968	0.241281228255258
USP1	0.0518560750600435	0.305730960023344
XRCC2	0.327489843101165	0.590117842472672
XRCC3	0.158255482480752	0.385286525554982
