# Supplementary figures and images for: Expression Profiling in Ovarian Cancer Reveals Coordinated Regulation of BRCA1/2 and Homologous Recombination Genes
Source: Biomedicines. 2022 Jan 18;10(2):199. doi: 10.3390/biomedicines10020199 (PMC8868827; doi:10.3390/biomedicines10020199)

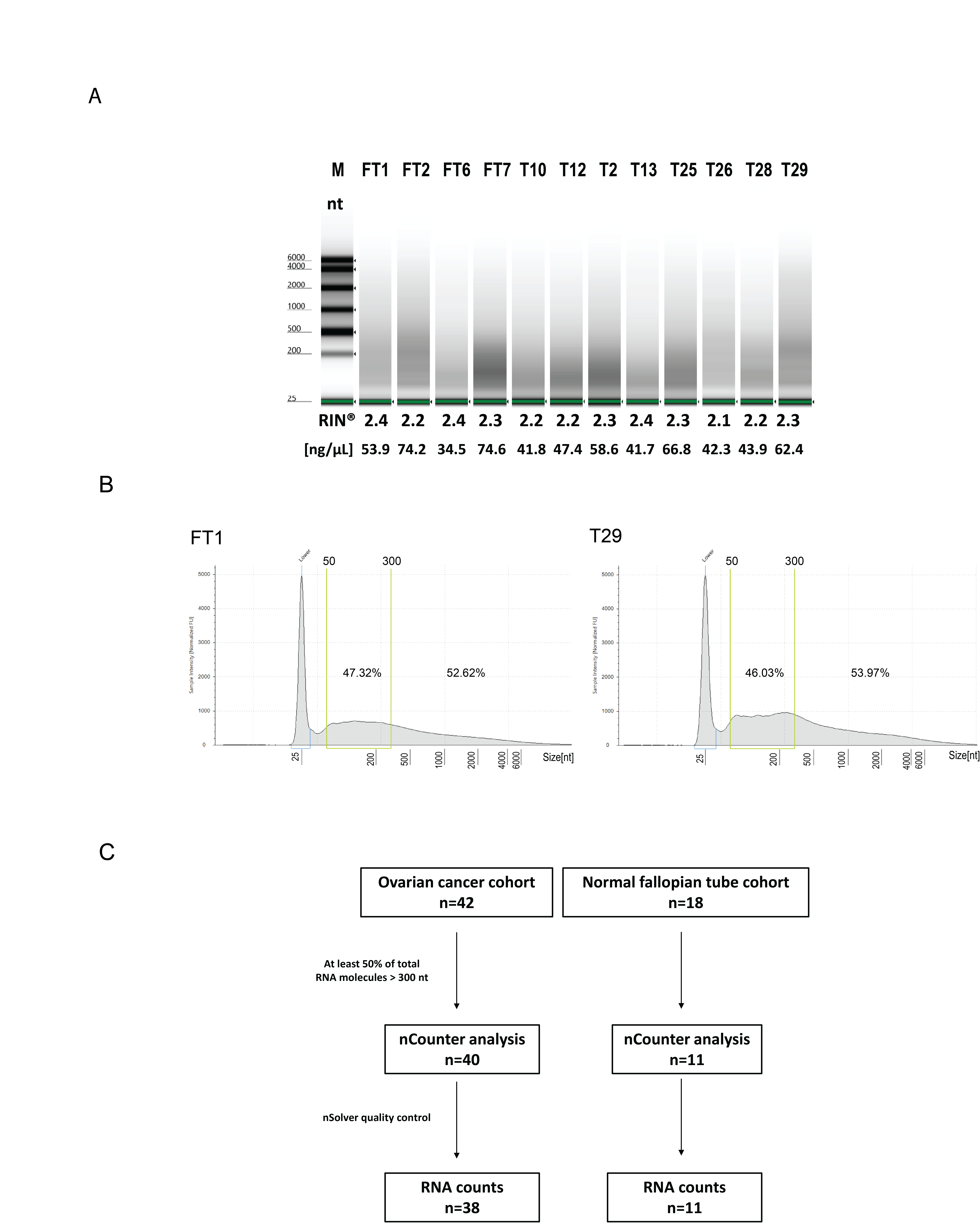

Supplement: Supplementary file 1 [file biomedicines-10-00199-s001.zip › Figure S1.tif]

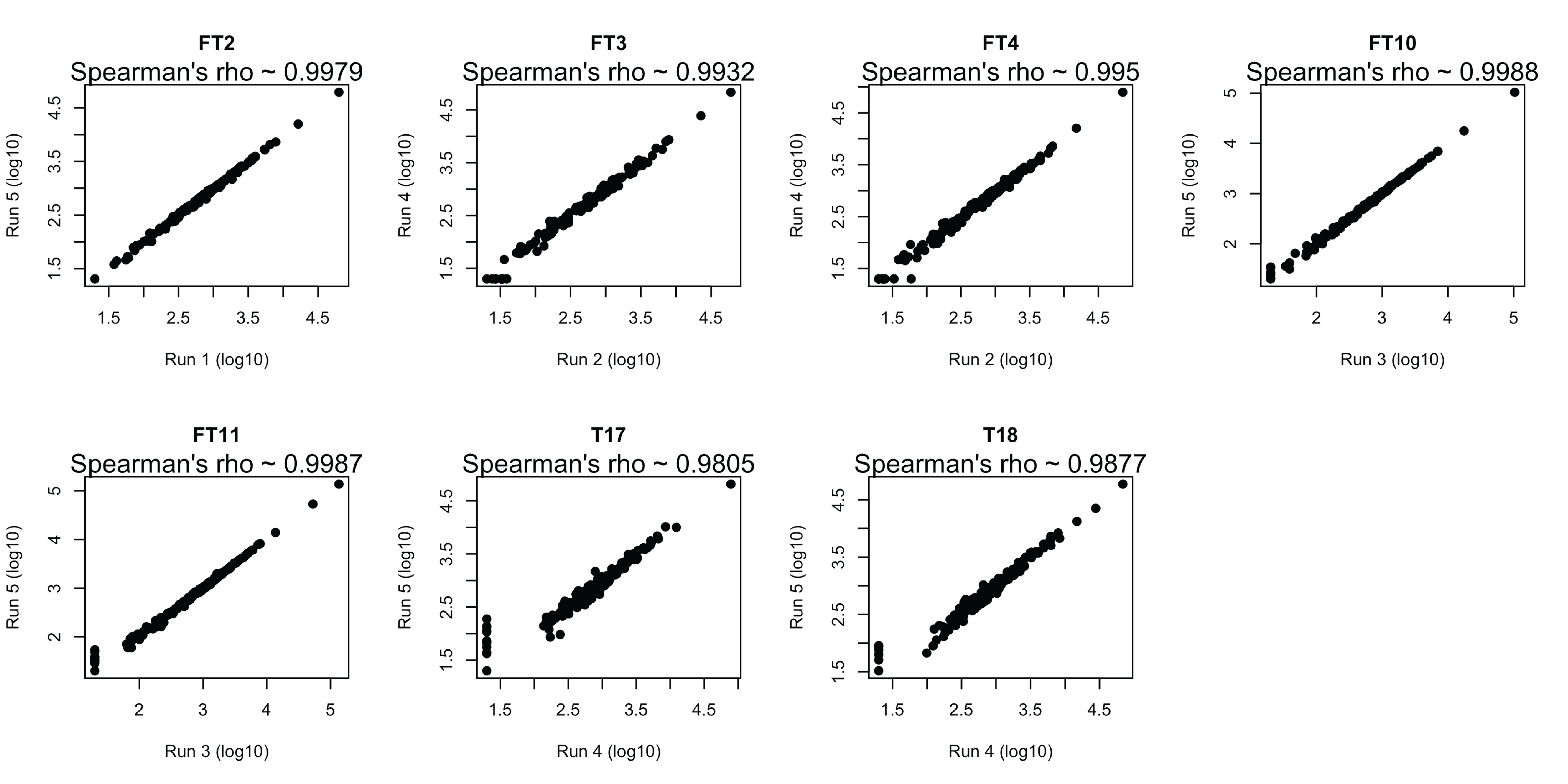

Supplement: Supplementary file 1 [file biomedicines-10-00199-s001.zip › Figure S2.tif]

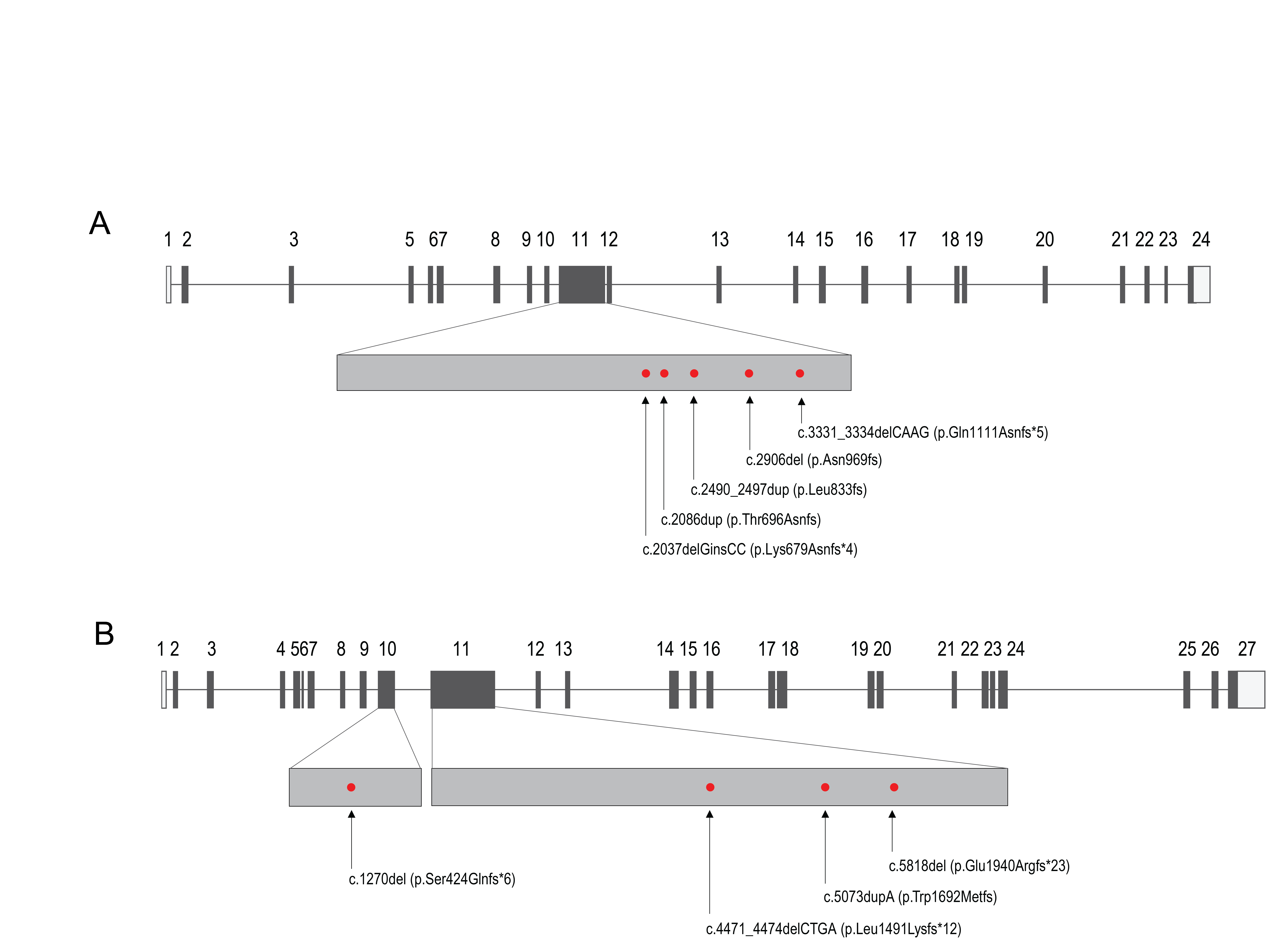

Supplement: Supplementary file 1 [file biomedicines-10-00199-s001.zip › Figure S3.tif]

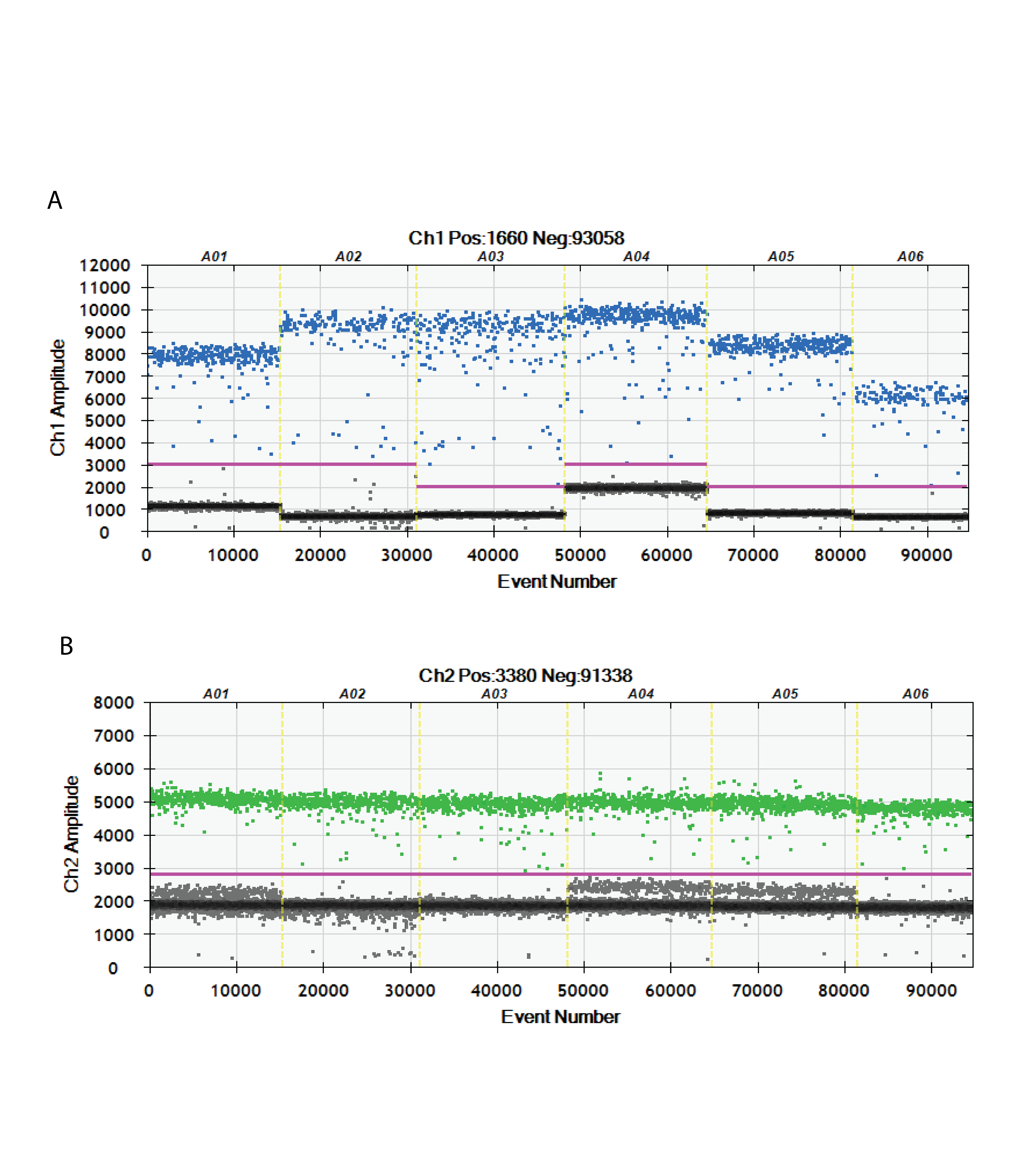

Supplement: Supplementary file 1 [file biomedicines-10-00199-s001.zip › Figure S4.tif]
